# What Does Temporal Brain Signal Complexity Reveal About Verbal Creativity?

**DOI:** 10.3389/fnbeh.2020.00146

**Published:** 2020-08-27

**Authors:** Yadwinder Kaur, Guang Ouyang, Werner Sommer, Selina Weiss, Changsong Zhou, Andrea Hildebrandt

**Affiliations:** ^1^Department of Psychology, Carl von Ossietzky Universität Oldenburg, Oldenburg, Germany; ^2^The Laboratory of Neuroscience for Education, The University of Hong Kong, Pok Fu Lam, Hong Kong; ^3^Institut für Psychologie, Humboldt-Universität zu Berlin, Berlin, Germany; ^4^Department of Individual Differences and Psychological Assessment, Institute of Psychology and Education, Ulm University, Ulm, Germany; ^5^Department of Physics and Centre for Nonlinear Studies, Institute of Computational and Theoretical Studies, Hong Kong Baptist University, Kowloon Tong, Hong Kong

**Keywords:** verbal creativity, divergent thinking, multiscale entropy, temporal complexity, individual differences

## Abstract

Recent empirical evidence reveals that creative idea generation builds upon an interplay of multiple neural networks. Measures of temporal complexity yield important information about the underlying mechanisms of these co-activated neural networks. A few neurophysiological studies investigated brain signal complexity (BSC) during the production of creative verbal associations and resting states, aiming to relate it with creative task performance. However, it is unknown whether the complexity of brain signals can distinguish between productions of typical and original verbal associations. In the present study, we investigated verbal creativity with multiscale entropy (MSE) of electroencephalography (EEG) signals, which quantifies complexity over multiple timescales, capturing unique dynamic features of neural networks. MSE was measured in verbal divergent thinking (DT) states while emphasizing on producing either typical verbal associations or original verbal associations. We hypothesized that MSE differentiates between brain states characterizing the production of typical and original associations and is a sensitive neural marker of individual differences in producing original associations. Results from a sample of *N* = 92 young adults revealed slightly higher average MSE for original as compared with typical association production in small and medium timescales at frontal electrodes and slightly higher average MSE for typical association production in higher timescales at parietal electrodes. However, measurement models failed to uncover specificity of individual differences as MSE in typical vs. original associations was perfectly correlated. Hence, individuals with higher MSE in original association condition also exhibit higher MSE during the production of typical associations. The difference between typical and original association MSE was not significantly associated with human-rated originality of the verbal associations. In sum, we conclude that MSE is a potential marker of creative verbal association states, but replications and extensions are needed, especially with respect to the brain-behavior relationships.

## Introduction

### Creativity as a Complex Trait

Creativity is a complex construct, defined as the process of producing an original and appropriate outcome (Mumford et al., 2003; Runco and Jaeger, 2012; Gabora, 2019). Creative solutions require complex thinking processes, such as divergent thinking (DT; Guilford, 1950). The creative process includes various aspects, such as fluent idea production, flexibility of thought, degree of elaboration, and originality of ideas (Guilford, 1956; Guilford and Merrifield, 1960). Previous research often focused on the study of fluency, that is, the ability to produce many ideas in a short amount of time, and originality, characterizing the quality of an idea. The answers given in DT tasks are mainly evaluated by humans (Silvia et al., 2008), especially when it comes to judging the product of a creative thought process in terms of its originality. Thus, human ratings of task outcomes are also customary in neuroscience (see Fink and Neubauer, 2008).

In general, healthy and functional biological systems are highly complex resulting from the long process of evolution and self-organization (Lewis, 2000, 2005). Advancement of functions or emergence of new functions is, thus, associated with increased system complexity during the evolution process. It has been proposed that the human brain is an adaptive system where highly complex neural networks may produce similarly complex psychological states and activities, such as consciousness and creative thought (Laycraft, 2009).

In a similar vein, the Honing theory (HT) of creativity proposed by Gabora (2017) links complexity-related concepts with creativity, suggesting that human minds are self-organizing, self-maintaining, and self-producing complex systems that subserve creativity. More concretely, the central idea of HT is that our minds evolve through an adaptive self-organization process in response to unpredicted (novel) environmental inputs, leading to a state of psychological entropy (Hirsh et al., 2012). This entropic state fosters creativity and aims to return to an equilibrium for further adapting to the environment. Hirsh et al. (2012) described psychological entropy as anxiety-provoking uncertainty, whereas Gabora (2017) redefined this assumption by replacing anxiety with arousal, conceptualizing creativity as a process of managing the state of psychological entropy in a positive sense. Empirically, this idea is supported, for example, by the fact that creative individuals exhibit greater openness to experience and higher tolerance to ambiguity (Feist, 1998).

Honing theory seeks to explain how ideas evolve over time considering the brain as a self-organizing complex system, which continuously interacts with and adapts to the environment to minimize psychological entropy. The theory aims to illustrate that psychological entropy is a driver of creativity impelled by emotions and intuitions (e.g., Cropley, 2006; Gabora, 2017) that plays a key role in monitoring and tracking creative progress. Following a similar line of theorizing, in the present research, we propose that the concepts of HT can be applied to understand the temporal complexity of EEG signals during creative verbal associations. More concretely, we assume that the challenge of solving a DT task applied in a laboratory setup will increase psychological entropy, which will be reflected in the brain signal during the time of dealing with this challenge.

Thus, in analogy to the HT aiming at explaining the creative process on a larger timescale across human evolution, in the present research, we focused on production of creative verbal associations at shorter timescales, defined as the time of generating a specific idea in response to a laboratory EEG task. To this aim, we adapted a well-established verb generation task (from Prabhakaran et al., 2014), requiring to produce a verb that is semantically related to a presented noun. This task is easy to administer despite the constraints of neural data acquisition. It was originally designed to evoke brain activity associated with semantic processing (Petersen et al., 1989) but was modified to assess creative verbal association production. In general, creative verbal production is a well-investigated instance of creativity. Therefore, we manipulated psychological entropic states by asking individuals to produce answers in two conditions: either original (by making original verbal associations) or typical associations (by recalling the first verbal association that comes into mind). EEG has been widely and fruitfully applied in various creativity studies to capture the complex and transitory brain activity during creative idea generation. Stevens and Zabelina (2019) reviewed creativity studies that used EEG and summarized its advantages to assess fast-moving and complex brain activity during the creative process. In the here applied task paradigm, we expected to differentiate the two creative task conditions at the neural level in terms of temporal complexity of the EEG signal. We further postulate that the EEG-captured brain signal, recorded while an individual generates original associations, will differ from the signal during states of generating merely typical associations. Therefore, EEG complexity should be higher during original verbal association states.

### Verbal Creativity and Brain Signal Entropy

In human brain cortical areas are interconnected by numerious neuroanl connections which form specialised neuronal networks. These networks are characterised by complex non-linear dynamic patterns. The interaction between various excitatory and inhibitory reentrant loops in these networks cause transient fluctuations in the brain signals over time, such as synchronous oscillatory activity (Friston and Price, 2001). Such transients are believed to reflect transitions between network microstates that can be used as an estimate of complexity underlying the network. Hence, greater variability in the amplitude pattern of the signal over time indicates a more complex system (Deco et al., 2011; Heisz et al., 2012). Healthy brain functioning has been characterized by two key components, variability and complexity of neural signals. The variance in neuroimaging time series data or neural signal variability has been suggested to be a proxy indicator of the neural dynamic characteristics, cognitive performance, and even brain disorders (Garrett et al., 2011; Garrett et al., 2013). In a similar vein, BSC has been explored as a possible neural correlate of cognitive performance. Entropy-based methods have been also used to examine brain signal variability and complexity, aiming to establish relationships with creativity. For example, Shi et al. (2019) used entropy measures of fMRI data to characterize the resting-state temporal dynamics and found a small-to-moderate positive association with verbal creativity. Sun et al. (2019) reported a correlation between verbal creativity and the temporal variability of functional connectivity patterns in the control network. In a similar line of research, a BSC measure known as MSE has been considered a potential EEG correlate of creativity. MSE is an information theoretic metric that provides an index of network complexity across multiple spatiotemporal scales (Costa et al., 2002, 2005). It uses sample entropy (SampEn) to quantify the irregularity of a time series at each of several scales achieved by coarse graining the original signal. A study by Ueno et al. (2015) showed higher MSE in resting-state EEG across large temporal scales in more creative as compared with less creative elderly individuals. Given the limited number of studies showing an association between verbal creativity and brain signal variability/complexity, we intended to further investigate this intriguing association by assuming that MSE can serve as a neural marker of verbal creative performance assessed with a DT task in younger individuals.

### Global and Local Neurophysiological Explanations of Creativity

Recent neuroimaging studies have allowed a better understanding of network dynamics and brain regions involved in creative ideation. Abraham (2018) summarized and divided the current state of knowledge on the neurophysiological basis of creativity into global- and local-based explanations. Global explanations view creativity as being grounded on large and widespread systems in the brain. According to these explanations, creativity is not composed of one but a series of multiple, simultaneously operating processes. Thus, the complex trait of creativity emerges from large-scale neural assemblies working in synchrony during the time of heightened creativity. In this line, a review by Beaty et al. (2019) elaborated on the creative network dynamics and demonstrated that the executive and default mode networks can reliably predict creative thinking ability of individuals. They argued that creativity is a result of the interaction between associative and executive processes. A functional connectivity study by Beaty et al. (2018) revealed that creative ability was associated with activity in interacting brain regions including the default mode, central executive, and salience networks, supporting the broad network view of creativity. A meta-analysis of functional imaging findings on creativity by Gonen-Yaacovi et al. (2013) identified a set of frontal and parieto-temporal regions activated during tasks that engage creative thinking. Local explanations of creativity focus on elucidating the specific brain regions involved in creative cognition, which have shown distinct contributions of the prefrontal cortex (PFC; for a review see Dietrich and Kanso, 2010). Frontal areas such as the Brodmann area 10 (BA 10) is regarded as an integrator of the output of many cognitive operations (Ramnani and Owen, 2004; Abraham, 2018). The BA 10 has been shown to be active during creativity tasks that require the integration of weakly related concepts during creative idea generation, conceptual expansion, musical improvisation, and analogical reasoning (Abraham et al., 2012b; Beaty, 2015; Abraham, 2018). Furthermore, lesions in the PFC have been associated with low performance in many creative cognition tasks, such as fluency and originality (see Abraham et al., 2012a). Additionally, ventrolateral and dorsolateral PFC areas located posterior to the frontal pole were shown to be involved in creative story writing and conceptual expansion, as well as in processing metaphors (Abraham et al., 2012b; Kröger et al., 2012; Gold et al., 2012). Thus, our hypotheses in the present study are built upon a global view on creativity, which we approach by using multiple timescales explicitly indicating spatial interactions in neural systems and not only temporal ones (Liu et al., 2019). Local explanations of creativity are reflected in our approach by as we specifically focus on prefrontal brain activity.

### Aims of the Present Research

Building upon the theoretical views and empirical evidences reviewed above, in the current study, we explored verbal creative word generation as an integrated activity of widely distributed but predominantly prefrontal neural networks. To this aim, we applied MSE analysis that has been proposed to quantify temporal complexity in EEG signals. MSE parameterizes the complexity of temporal patterns underlying any kind of time series. When applied to brain signals, MSE provides information reflecting the communication of different neural generators in functional brain networks across multiple timescales (Heisz and McIntosh, 2013). From a theoretical point of view, small timescales in MSE reflect local neural interactions, while large timescales reflect activity of widely distributed neural networks (Grundy et al., 2017; Vakorin et al., 2017). Linear stochastic effects are assumed to be related to observational noise at lower timescales. Coarse-graining applied during MSE analysis (see “Materials and Methods” section for details) is essentially a down-sampling process, which alleviates linear effects in large timescales. Thereby, small timescale MSE extracts information from the whole frequency spectrum and also captures linear stochastic effects in the signal, while large timescale MSE relates to slow oscillations and reflects non-linear signal properties (Courtiol et al., 2016; Miskovic et al., 2019). Therefore, by applying MSE to EEG signals recorded during typical vs. original associations, we aimed to capture the stochastic properties of the EEG signals that are assumed to be associated with the joint neural activities of local (small scales) and widely distributed (large scales) brain networks. We thus interpret activity of broadly distributed networks on the basis of MSE at large timescales.

Assuming MSE to be a neural marker of creative cognition, we hypothesized (1) a quantitative MSE difference, in the sense that efforts to produce original verbal associations will lead to higher average MSE as compared with typical verbal associations. (2) We expected brain states during the production of original associations to qualitatively differ from brain states during typical association production, which might be reflected in specific rank orders of individuals with respect to their MSE in these two states. (3) We further expected the stronger MSE difference between typical and original associations to especially occur at frontal areas. (4) We aimed to explore whether the MSE difference between typical and original association states is associated with performance in terms of originality ratings of the produced associations.

## Materials and Methods

### Participants

The sample of the present study consisted of *N* = 101 participants (51 females). In the following steps, we merged the behavioral data (human-rated originality scores of verbs) with the EEG acquired during verb associations. We excluded eight participants with less than 10 years of German language-speaking experience and one case of invalid EEG event markers. Thus, the final sample included *N* = 92 participants (43 females, *M*_age_ = 23.88, range = 18–32 years); 89 individuals were native German speakers; 8 had not obtained high school degrees, 67 had high school or equivalent degrees, and 17 had academic degrees (e.g., bachelors, masters, or diploma).

### Neurophysiological Recordings

Electroencephalography datasets were recorded in a closed, quiet, and well-illuminated room using the Brain Vision Recorder software (Brain Products, Germany). The EEG signals were amplified using BrainAmp DC amplifiers (Brain Products, Germany) with an amplitude resolution of 0.1 μV. We used 0.16 and 1,000 Hz as low and high cutoff filters, respectively, and a sampling rate of 250 Hz. An EEG cap (Easycap, Brain Products, Germany) was mounted with 30 Ag/AgCl electrodes, placed according to the 10–20 system. Eye movements and blinks were monitored with electrodes positioned at the outer canthi of both eyes and below the right eye. The A1 electrode (left mastoid) was used as online reference, and AFz served as ground. Impedances were kept below 5 kΩ.

### Preprocessing of Electroencephalography Data

Offline, the EEG signals were filtered using IIR (zero phase shift) and Butterworth filters between 0.1 and 50 Hz (order = 2; time constant = 1.59 s) and recalculated to average reference using Brain Vision Analyzer (Brain Products, Germany). Further preprocessing steps were executed in EEGLAB (Delorme and Makeig, 2004); SASICA (EEGLAB plugin; Chaumon et al., 2015) was used to remove eye blinks, movement, and electro-cardiac artifacts. We applied SASICA on the basis of autocorrelation measures and focal topography. Noisy components like muscle movements tend to show low autocorrelation. Therefore, muscle artifacts were detected by measuring the time-point by time-point variability, which was captured by low autocorrelation measures. Tonic muscle artifacts were detected based on their noise patterns and focused topography on electrodes around the edge of the EEG cap. Since the time window for the MSE analyses was defined from the onset of the stimulus until the onset of the participant’s typing response (see Supplementary Figure S1), the probability of muscle movement artifacts during this interval was very low.

### Tasks and Procedure

**FIGURE 1:**
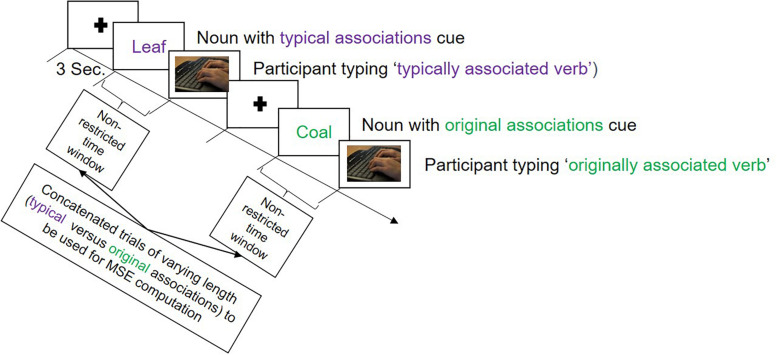
Illustration of the trial sequence in the verb generation task employed during the electroencephalography (EEG) recording session. The task began with a fixation cross presented for 3 s, followed either by a purple colored noun, called ‘typical associations cue,’ to which participants should produce a commonly known association, or by a green colored noun called ‘original associations cue,’ to which participants were expected to produce an original association. There were no time limits for the responses.

In the verb generation task, there were two types of color-cued nouns presented: purple and green. To purple-cued nouns, participants were expected to produce typical associations—we thus instructed them to type in the verb that first came to their mind when being presented with the noun. To green-cued nouns, participants should produce original, unique verb associations in response to the noun (see Figure 1). We modified the original task by translating the stimulus material (adapted from Prabhakaran et al., 2014) into German and dropping some nouns that were not proper in the German language (see Supplementary Material S1 for the list of original English nouns and their German translations with additional explanations for dropped trials). This resulted in 35 purple- and 32 green-cued nouns, signaling the production of typical and original associations, respectively. The task started with verbal instructions followed by an example trial and five practice trials. Participants were instructed to type in only one associated verb for each presented noun. The onset of the stimulus and the onset of participant’s typing response were time-marked, to be taken as signals of interest for MSE analysis. There were no time limits during the experimental trials in order to capture the brain activity during the complete creative verbal association production. PsychoPy (Peirce and MacAskill, 2018) was used to present the stimuli and record the behavioral data. EEG was recorded during the entire task, which lasted for ∼20 min, depending to some extent on the participant.

### Human Ratings of the Verb Production Task Outcomes

**FIGURE 2:**
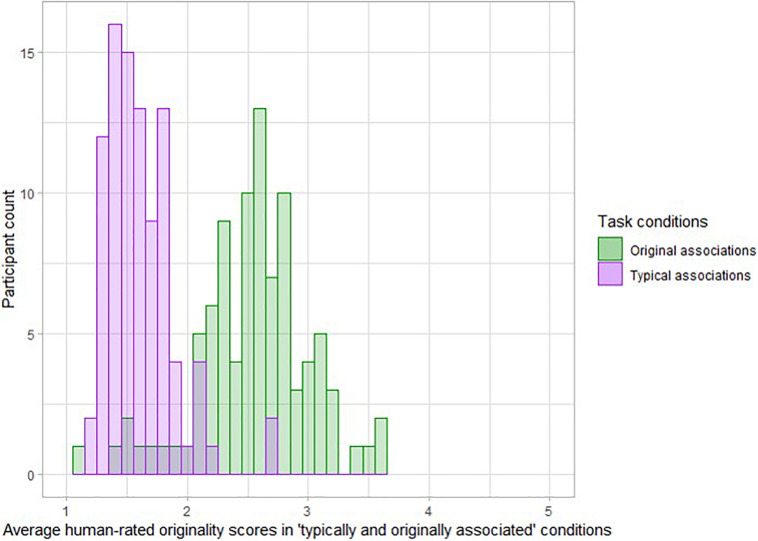
Distribution of average rated originality obtained across the condition-specific items of the verb generation task, across participants. Taken together, the histograms illustrate the participants more often produced original associations in the condition in which original associations were expected.

Three trained native German speakers rated all verbs produced during the task for originality. The raters were aware of the condition of origin of each verb but were instructed to rate the originality of the provided answer without taking the condition into account. The originality was assessed on a scale from 1 (not at all original) to 5 (unique and original), according to subjective scoring guidelines usually deployed in DT tasks (Amabile, 1982; Silvia et al., 2008). Such scoring guidelines usually explain that a highly original answer is an answer that is rare in the sample, remote from the presented noun and somehow unexpected for the rater (Silvia et al., 2008). Raters were instructed to use the total range of the scale if possible and to rate the generated verbs in relation to the answers provided by other participants. The intraclass correlations (ICCs; Shrout and Fleiss, 1979) across a fixed set of raters for all items ranged from 0.81 to 0.97. Due to this sufficient/good agreement between the three raters, we used an average score across all three raters per item for statistical analyses (see Figure 2). We label human ratings of the verb production task outcomes in the entire manuscript as “human-rated originality scores in typical associations and original association’s condition.”

### Multiscale Entropy Algorithm

**FIGURE 3:**
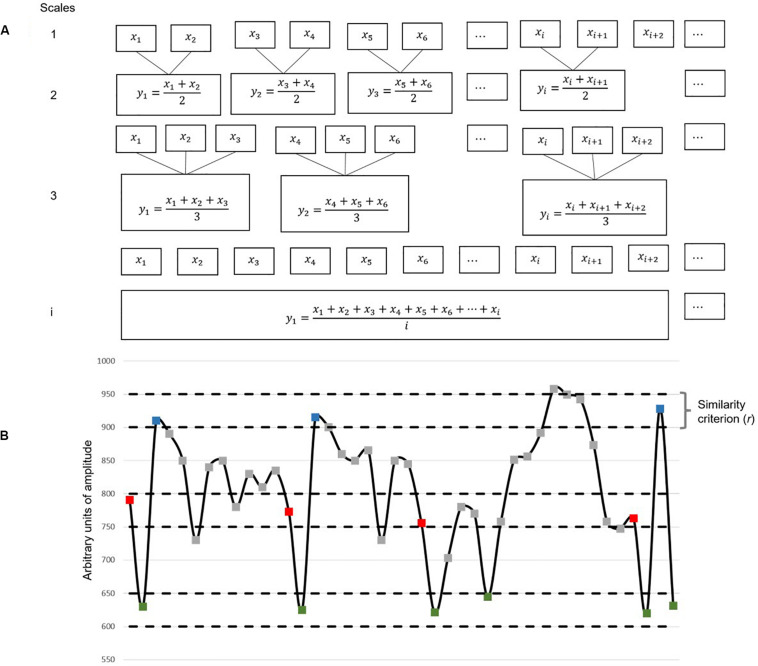
Illustration of multiscale entropy (MSE) algorithm. **(A)** Shows the first step of coarse-graining. Each box represents a data point. **(B)** Illustrates the second step in which the Sample Entropy (SampEn) of each coarse-grained time series is calculated (*m* = 2). This figure is an example with arbitrary data unit. In this example, with respect to the first two-point sequence (the red–green dyad), there are three other two-point sequences that are identified as similar patterns based on the threshold *r*. With respect to the first three-point sequence (the red–green–blue triad), there are two other three-point sequences that are identified as similar patterns based on *r*. Likewise, the algorithm will count the number of similar triad pairs (N3) and the number of similar dyad pairs (N2) from the entire sequence. SampEn is the natural logarithm of N2/N3.

We calculated MSE, following Costa et al. (2002, 2005), in two steps. (1) We first coarse-grained the original signal at different timescales—a procedure similar to low-pass filtering. The coarse-grained time series at timescale 1 is identical to the original signal; for obtaining scale n, the time series was divided into non-overlapping concatenating windows, each of which contains n points where n is the corresponding scale. Within each window, all data points were averaged, forming a new coarse-grained time series at that scale (for illustration, see Figure 3A). (2) SampEn was then calculated for each of these coarse-grained time series (Figure 3B). SampEn characterizes the entropy of a time series by calculating the recurrence probability of a specific dynamic pattern. Specifically, SampEn identifies repetition of sequence pattern in the time series and calculates entropy in three steps, as follows: (i) first, the number of sequences with *m* data points satisfying the similarity criterion are counted and denoted as *N*(*m*); (ii) the number of similar sequences with *m* + 1 data points length are counted and labeled as *N*(*m* + 1); and (iii) in the last step, SampEn is calculated as the negative natural logarithm of the conditional probability that two similar sequences of *m* data points will be similar for the next *m* + 1 points.

$$\text{SampEn}\,\left(m\right)=-\ln\frac{N\,\left(m+1\right)}{N\,\left(m\right)}$$

The two sequences are similar, if the difference between every point in the first sequence [*N*(*m*)] vs. the corresponding point in the second sequence [*N*(*m* + 1)] are less than *r* (see Figure 3). There are two critical parameters in SampEn calculation: *m* (pattern length) and *r* (similarity criterion). We adapted the conventionally used parameter settings, *m* = 2 and *r* = 15% of the SD of the original time series.

### Mapping the Multiscale Entropy Timescales to Real Time

Multiscale entropy timescales (ranging from 1 to 20 in our study) can be mapped to real time. For example, according to the sampling rate (250 Hz) used in the present research, the real-time sampling interval at scale 1 is 4 ms. Therefore, MSE at scale 1 reflects dynamical activities of the neural system at a resolution of 4 ms, which is fast dynamics. In a similar vein, scale 5 reflects dynamical activities of the brain at a resolution of 20 ms, and scale 10 indicates activity at 40-ms resolution. At the highest scale 20, the activity is at 80-ms resolution, which reflects slow brain dynamics. Thus, at smaller timescales, MSE reflects fast and, hence, local neural activities, whereas at larger scales, MSE captures slow dynamics across broader spatial domains.

### Multiscale Entropy During Production of Typical and Original Associations

The trial length of the EEG recorded during the production of typical and original associations varied from trial to trial and person to person. The variation was inherent to the non-restricted response time (see Supplementary Figure S1, visualizing the average reaction times across all trials of “typical” and “original” association conditions across all participants; the figure shows that participants took a variable amount of time to provide their answers). For this reason, a decision had to be made whether to consider trial-to-trial and person-to-person variable trial lengths for MSE analysis or to standardize the analysis interval across individuals. To empirically substantiate this decision, we systematically explored whether the average MSE for both varying trial length (from noun presentation to response) and standardized trial length (for which we fixed the trial length from noun presentation to a frame of max. 4 s for each participant) would differ in terms of individual differences (see results and analysis in the Supplementary Material S2 illustrating the correlation between the two options of analyzing MSE, based on variable vs. standardized trial length). Pearson correlation matrices of MSE, analyzed in four concatenated trial segments of standardized vs. varying length, indicated very high associations between the two. Thus, the rank order of individuals barely differs when estimating MSE from standardized vs. varying trial lengths. Hence, the decision can be made according to theoretical considerations. Note that we aimed to capture the complete idea generation process, and the variable length covering the whole thinking time is a more appropriate option from this theoretical point of view. Thus, for the final MSE analysis, the trial segments with variable length were concatenated for each condition and participant. The concatenated segments were further divided into four data segments to be used as indicators for the structural equation modeling (SEM). The length of the concatenated signal segments did not vary within participant and across timescales; only the resolution did. Due to progressive coarse graining (from sampling interval 4 ms at scale 1 to 80 ms at scale 20), which lies at the heart of MSE calculation, the resolution of the signal differs across timescales. But the analyzed trial length varied across individuals.

To summarize, we calculated MSE for each participant, at each channel (electrode) across multiple timescales (ranging from scale 1 to 20) in 2 (conditions) ^∗^ 4 data segments (for adjusting unreliability of MSE estimates and for conducting statistical inference at the level of latent variables).

### Statistical Analysis

Multiscale entropy difference tests between conditions and brain-behavior associations during the creativity task (human-rated originality scores of the generated verbs) and MSE estimates were conducted by means of SEM. SEM is a generalized linear modeling framework proposed as a combination of confirmatory factor analysis and path modeling. For an introduction to SEM, we refer to Kline (2015). SEM with latent variables has the great advantage that a measure (dependent variable) can be decomposed onto (1) the true score, (2) its method or content specificity, and (3) its measurement error. For the present endeavor, by means of SEM, measurement error (unreliability arising due to the estimation noise of MSE across different data segments) can be accounted for, prior to inferentially testing mean differences between experimental conditions. Furthermore, the SEM approach allows directly investigating the correlation between MSE captured in different experimental conditions and their difference with behavioral outcomes. Finally, with an SEM approach, integrated measures can be used to avoid multiple testing issues.

Note that a measurement model requires a minimum of four indicators for a latent variable to be identified. Therefore, MSE was computed in four different segments as described above. Calculating condition-specific latent MSE variables with four indicators (latent variable for MSE during typical and original association) will thus allow to test hypotheses at the level of latent variables, which are corrected for measurement error. More importantly, by using latent variables, we can jointly test hypotheses with respect to mean differences and individual differences. In summary, we used SEM to quantify mean and individual differences in two different conditions of creative verbal associations, and we investigated the latent level relationship between MSE measures in the two conditions to make inferences about the specificity of individual differences.

Statistical analyses were performed with the R Software for Statistical Computing (R Core Team, 2018). For SEM estimation, we used the lavaan (LAtent VAriable Analysis) package by Rosseel (2012). We evaluated model fit by the following test statistics and fit indices: the chi-square fit statistic (χ^2^), the comparative fit index (CFI, that should exceed 0.95 for a good fit), standardized root mean square residual (SRMR; to be lower than 0.08), and root mean square error of approximation (RMSEA to be lower than 0.08); please see Kline (2015) for more information about SEM fit.

## Results

### Descriptive Multiscale Entropy Results

**FIGURE 4:**
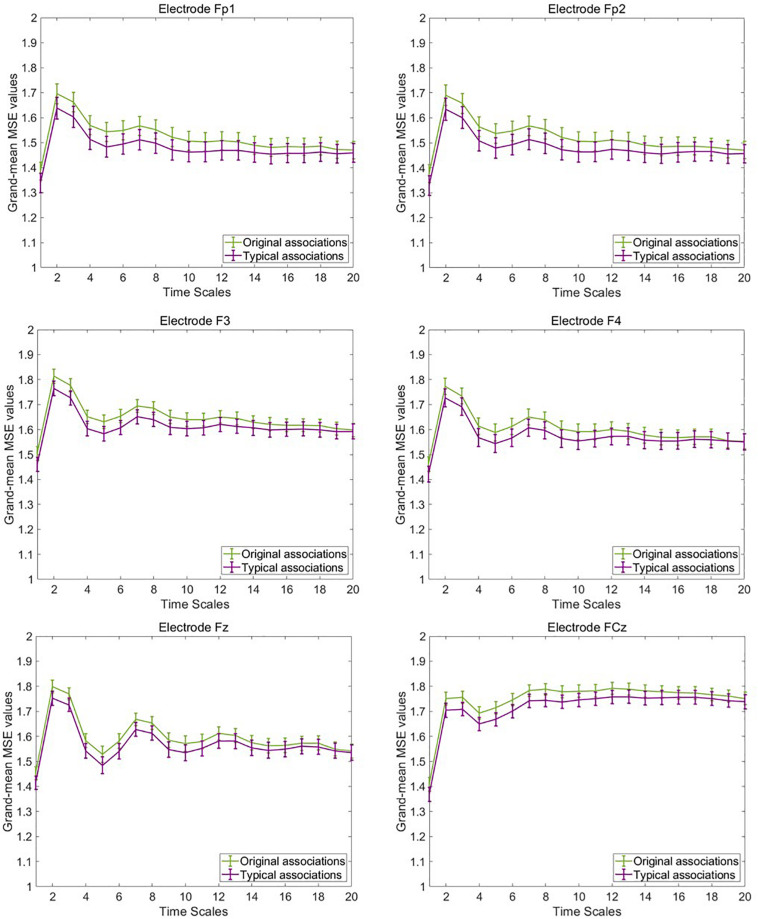
Grand-mean multiscale entropy (MSE) during typical and original associations at frontal electrodes across 20 time scales. The MSE is slightly larger in the originality condition mostly at frontal electrodes at small (1–5) and medium (6–15) time scales. Error bars represent 1 SE.

To illustrate mean MSE differences at the observed level between typical and original associations, we computed the grand-mean MSE across the four segments of concatenated EEG trials and participants separately for both experimental conditions, each electrode, and timescale. Figure 4 provides line plots with error bars of grand-mean MSE during original associations (green line) and typical (purple line) associations at six frontal representative electrodes. Error bars represent standard errors. Note that we do not aim to conduct statistical tests at this data level, which is not adjusted for measurement error. Descriptively, differences between the two conditions occurred especially at frontal electrode sites at small (scale 1–5) and medium (scale 6–15) timescales (see line plots for further electrodes in the Supplementary Material S3). These results suggest that a slightly higher complexity characterized the brain signals during production of original associations as compared with typical associations. The only statistical test conducted at this level was the one on sex differences to rule out potential confounders in the subsequent latent variable analyses. Results are provided in the Supplementary Figure S4, which suggest no sex differences in MSE within and between the task conditions.

**FIGURE 5:**
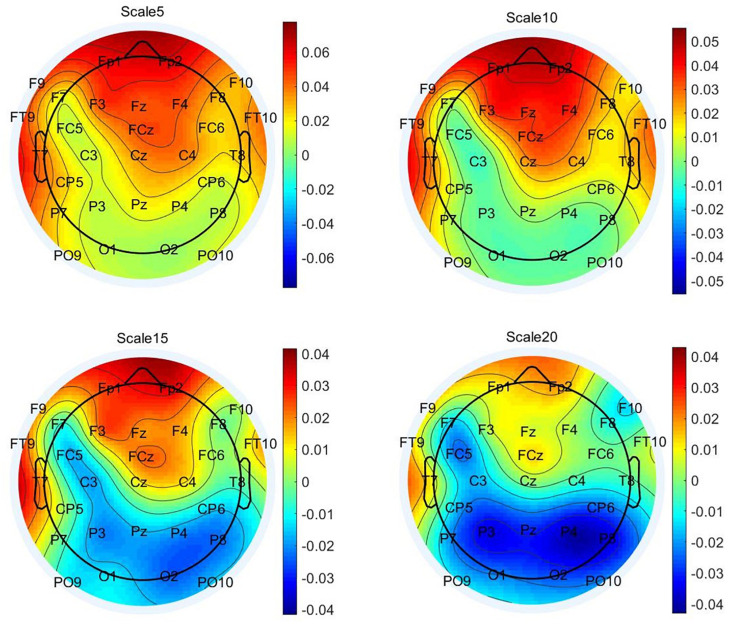
Topographic plots of grand-mean multiscale entropy (MSE) difference between original and typical associations at time scales 5, 10, 15, and 20. These distributions illustrate that the positive difference in MSE between the two idea generation conditions occurs at frontal regions and up to scale 15 and negative differences are prominent at parietal sites at Larger scales.

Next, we visually explored the topographical pattern of the MSE difference between the two experimental conditions at this observed data level. To this purpose, we calculated the absolute differences in mean MSE between the two conditions and obtained their scalp topographies at selected small, medium, and large timescales. Visual inspection of these plots reveals six frontal electrodes Fp1, Fp2, F3, F4, Fz, and FCz where the positive differences in MSE were the largest and P7, P3, Pz, P4, and P8 parietal electrodes where the MSE difference was negative (see Supplementary Material S5 for the topoplots at all remaining temporal scales). According to our theoretical expectations, frontal electrodes are of special interest for statistical testing at the level of latent variables. We thus selected the six frontal electrodes for latent variable analyses. Because these differences at observed level are not adjusted for unreliability, the illustration in Figure 5 fulfills, similarly to the line plots in Figure 4, a descriptive purpose only. For subsequent statistical tests, MSE measures were spatially and temporally integrated and adjusted for measurement error. Thus, an average across these six electrodes and integrated measures across temporal scales was considered (see below).

### Latent Mean and Individual Differences in Multiscale Entropy

**FIGURE 6:**
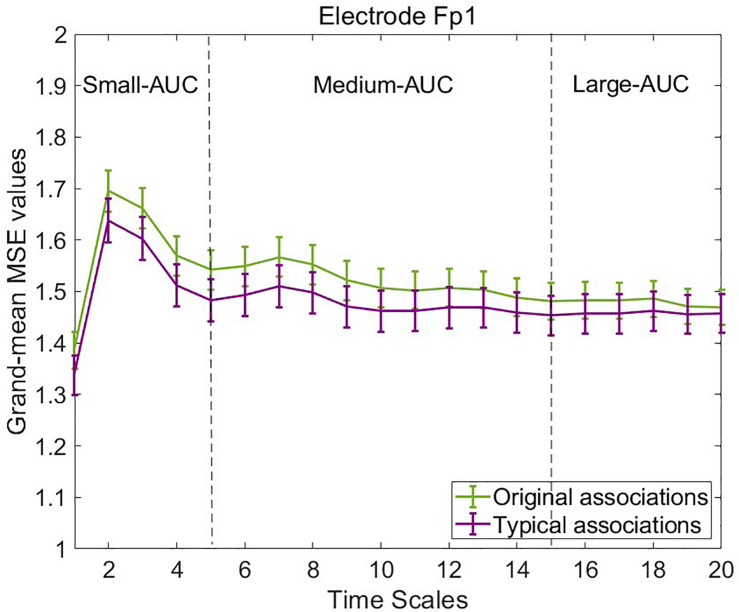
Illustration of the time scale integration using the area under the curve (AUC) measure. Small-AUC is obtained by summing up the multiscale entropy (MSE) values across time scales 1–5; Medium-AUC by integrating across scales 6–15; and Large-AUC is achieved by integrating MSE values across scales 16–20. Note that the magnitude of Medium-AUC values differs from the other two, because 10 as compared to 5 single values are summed up in that case.

**FIGURE 7:**
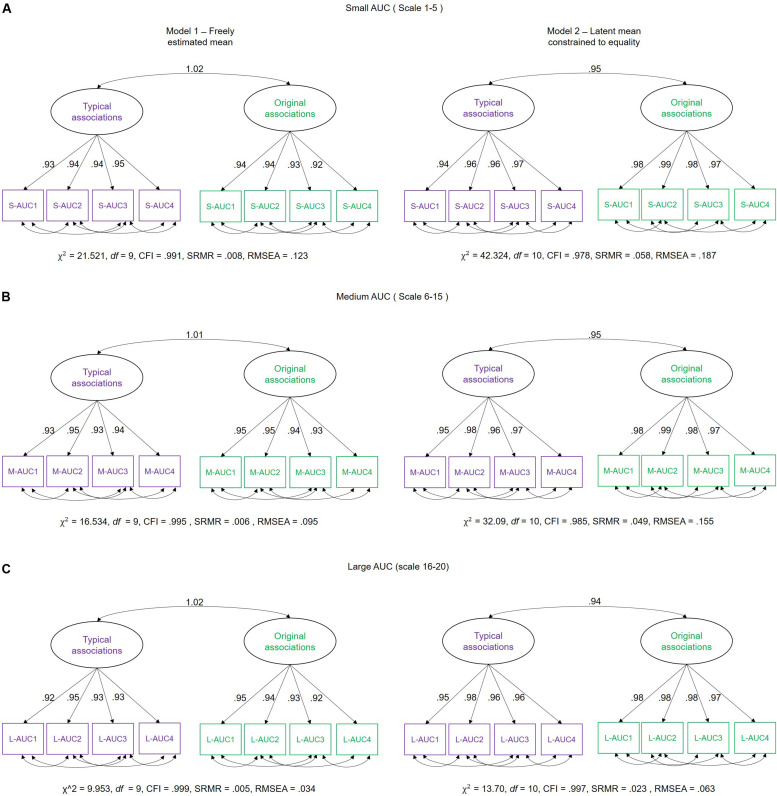
Schematic representation of measurement models investigating the difference between latent means and individual differences in multiscale entropy (MSE) as measured in typical and original associations. We estimated two models: Model 1 – in which the mean of the latent variables (typical and original associations) were freely estimated, and Model 2 – in which the means of the latent variables were fixed to equality. These models were separately estimated for **(A)** Small-AUC scores (integrated across smaller time scales 1–5), **(B)** Medium-AUC scores (integrated across medium time scales 6–15), and **(C)** Large-AUC scores (integrated across large 16–20 time scales). S-AUC (1–4) – first to fourth indicators of small AUC scores, M-AUC (1–4) – first to fourth indicators of medium AUC scores, L-AUC (1–4) – first to fourth indicators of large AUC scores. χ^2^, Chi-square; *df*, degrees of freedom; CFI, comparative fit index; RMSEA, root mean square error of approximation; SRMR, standardized root mean-square residual.

For the purpose of hypothesis testing, we estimated a two-factorial measurement model with correlated factors (see SEM description above), differentiating latent MSE variables for typical and original associations each. To avoid multiple testing, we integrated MSE values across several scales into single scores. This procedure has been previously proposed in the literature as an approach to handle such multiple scale measurements (see Takahashi et al., 2009; Kaur et al., 2019). Hence, we used the area under the curve (AUC) as an integrated entropy score per participant. Visual inspection of the line plots of Figure 4 suggests that the MSE difference between the two experimental conditions increases across small scales (1–5) shows a rather stable condition difference at medium scales (6–15) but no difference at large scales (16–20). Therefore, we divided the timescale-specific MSE values into three categories (small-scale MSE, ranging from scales 1–5; medium-scale MSE, including scales 6–15; and large scale, MSE from scale 16–20) and integrated the person- and condition-specific MSE values by summing them across those scales (see Figure 6 for a graphical explanation of this procedure). With the use of these AUC scores, separate measurement models for small, medium, and large scales were estimated (Figure 7). Using these models, we investigated whether latent condition-specific means are substantially different from each other or can be constrained to equality without significantly diminishing model fit (according to the χ^2^-difference test). If the model fit would substantially decrease by constraining the latent MSE means of the typical vs. original association conditions to equality, we would conclude that the mean difference is statistically substantial.

In Model 1, the mean of the latent MSE variables, i.e., typical and original associations, was freely estimated. In the Model 2, an equality constraint was set on the latent variables’ means. Results are displayed in Table 1, which shows the estimated means and the latent mean after imposing the equality constraint. For small AUC, the Δχ^2^ test showed that the equality constraint diminished the model fit significantly. For medium AUC, the model fit significantly diminished as well by constraining the means to equality, which was, however, not the case for large AUC. These results suggest that the modeled latent mean differences in MSE between the two conditions are statistically substantial for small and medium, but not for large, timescales. To provide an effect size estimate, we averaged across the indicators separately for small, medium, and large scales and used the formula for calculating the paired samples, repeated measures *d* coefficient. For small AUC, the observed average mean MSE during typical associations was 6.436 (*SD* = 1.207), whereas in original associations, it was 6.633 (*SD* = 1.109). Given a correlation of *r* = 0.957 between the repeated within-person measures, the effect size for small AUC amounts to *d* = 0.531. For medium AUC, the observed average mean MSE during typical associations was 14.271 (*SD* = 2.698), whereas in original associations, it was 14.608 (*SD* = 2.477). Given the correlation of *r* = 0.954 between the repeated within-person measures, the effect size for medium AUC amounts *d* = 0.414. For large AUC, the observed average mean MSE during typical associations was 6.284 (*SD* = 1.171), whereas in original associations, it was 6.346 (*SD* = 1.061). Given a correlation of *r* = 0.956 between the repeated within-person measures, the effect size for large AUC amounts *d* = 0.178. The *d* coefficients indicate a moderate effect size for small and medium scales and a negligible effect at large scales. Note that we provide effect size estimates for the manifest variables, because they are more conservative and because no clear guidelines exist for calculating *d* for latent variables.

**TABLE 1:** Model fit indices of the nested SEMs estimating latent mean differences in MSE between typical and original associations.

Timescales	Model	CFI	χ ^2^	*df*	Δχ ^2^	Δ*df*	*p*	Estimated mean MSE during original associations	Estimated mean MSE during typical associations	Mean MSE with equality constraint
Small AUC	1	0.991	21.521	9	–	–	–	6.729	6.457	6.744
	2	0.978	42.324	10	20.803	1	<0.001			
Medium AUC	1	0.995	16.534	9	–	–	–	14.730	14.210	14.769
	2	0.985	32.093	10	15.559	1	<0.001			
Large AUC	1	0.999	9.953	9	–	–	–	6.386	6.276	6.398
	2	0.997	13.700	10	3.747	1	>0.050			

*CFI, comparative fit index; χ^2^, chi-square; Δχ^2^, chi-square difference between Model 1 (freely estimated latent means) and Model 2 (equality constraints on latent means); Δdf, difference in the degrees of freedom between Model 1 and Model 2; SEM, structural equation modeling; MSE, multiscale entropy; AUC, area under the curve.*

To investigate the specificity of individual differences in MSE during typical vs. original associations, we examined the latent level correlation between the two latent variables in Figure 7. This is to ask whether individuals systematically differ with respect to MSE between the original and typical associations or whether the rank order of individuals is indistinguishable between the two conditions. Figure 7 illustrates the measurement models showing the relationship between the two latent variables for small-, medium-, and large-AUC scores. The latent level correlations are perfect (even abnormally estimated above the boundary of the correlation scale) when condition-specific means are allowed. Correlations are close to unity in each AUC (small, medium, and large AUC) when latent means were constrained to equality. Given these estimates, it can be concluded that individuals exhibiting higher (or lower) MSE in original associations are also characterized by higher (or lower) MSE during typical associations. Thus, the rank order of individuals is non-distinguishable with respect to MSE in the two verbal association conditions.

### Relationship Between Human-Rated Originality Scores and the Latent Mean Multiscale Entropy Difference Between Typical and Original Associations

**FIGURE 8:**
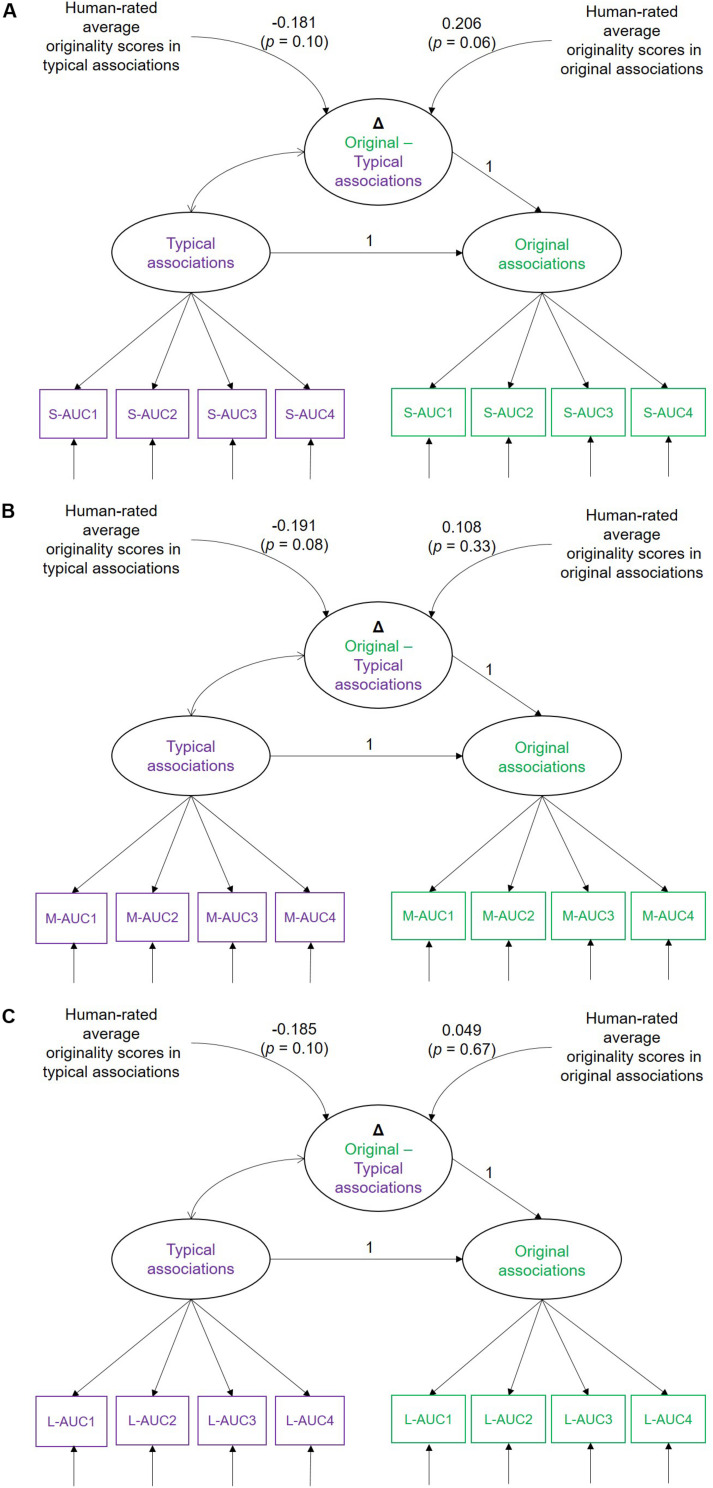
Simplified illustration of the latent difference score models (LDSMs) estimated for **(A)** small-AUC, **(B)** medium-AUC, and **(C)** large-AUC values of multiscale entropy (MSE). Typical and original associations are latent MSE variables indicated by four AUC values. Human-rated cross-trial average originality scores are obtained during the production of typical and original associations in the Verb generation task. Δ Original-Typical associations is the MSE difference score between the experimental conditions. S-AUC1-4, M-AUC1-4, and L-AUC1-4 are the first to fourth AUC indicators of the MSE values for small, medium, and large time scales, respectively.

To investigate how the MSE difference between typical and original associations is related with human originality ratings of the produced verb, we applied latent difference score modeling (LDSM; McArdle and Hamagami, 2001). Because observed difference scores are poor in their psychometric quality (lack of reliability and restricted variance; Raykov, 1999) and the covariance structure of the measurements is not taken into account when using them, relationships with behavioral outcomes were investigated with LDSM. LDSM parameterizes the absolute difference between two latent variables—that is, two experimental conditions in the present case. The estimated variance of the latent difference scores quantifies individual differences in condition effects. We estimated difference score models for small, medium, and large AUC and regressed them onto originality ratings of the verbs generated during production of typical vs. original associations. To test the hypotheses whether the MSE difference is larger at heightened originality values, the difference score was additionally regressed onto the squared originality ratings (quadratic effect, which was expected to be positive). Figure 8 schematically illustrates the difference score LDSMs, separately for small-, medium-, and large-AUC scores, including the linear terms only, because none of the quadratic effects turned out to be significant (see also Table 2).

**TABLE 2:** Results of the LDSM models.

			Regression Weight of the Difference Score Onto Human-Rated Originality Scores In	
		Intercept of		
Model	*SD* of difference score	Difference Score	Typical associations	Original associations
Small AUC	0.26	0.25	−0.18	**0.20**
Medium AUC	0.62	0.85	−0.19	0.10
Large AUC	0.26	0.33	−0.18	0.04

*Effect sizes above 0.20 are printed in bold. These models additionally included quadratic terms to test the hypotheses whether the difference between MSE in typical and original association is larger for heightened originality scores. The quadratic associations failed to reach statistical significance (regression weight of the quadratic term between human-rated originality score and the difference in MSE between typical and original association for small AUC = 0.024, *p* = 0.8; medium AUC = 0.009, *p* = 0.9; and large AUC = 0.008, *p* = 0.9). LDSM, latent difference score modeling; AUC, area under the curve; MSE, multiscale entropy.*

As illustrated in Table 2, individuals substantially vary in their MSE (absolute) difference scores between typical and original associations. This difference at small-scale MSE was positively associated with human-rated originality scores when original associations were expected. However, none of the further linear and quadratic associations were statistically significant.

Because the MSE difference between the two conditions was negative at parietal electrodes, we additionally performed statistical tests [the same as for frontal region of interests (ROIs)] for the parietal electrodes (P7, P3, Pz, P4, and P8). Note that these effects were not hypothesized. These exploratory analyses revealed no statistically substantial associations (see results in the Supplementary Material S6).

Thus, the present data, given the statistical power at hand, reveals no robust linear association between the MSE difference between typical and original association conditions and human-rated originality scores of the produced associations. However, with a larger sample size, we might find that individuals with higher temporal complexity in frontal sites when producing original associations tend to be more original.

## Discussion

The present study aimed to understand creative verbal association states at the neural level within the framework of complexity theories. We employed the MSE algorithm as a complexity estimate in neural signal during a verb generation task. As hypothesized, temporal complexity was higher during production of original associations as compared with typical verbal associations across small and medium timescales in frontal areas. However, the magnitude of this difference was small or moderate and statistically substantial only up to scale 15 (in the range of medium scales). The latent correlations between entropy as estimated in original vs. typical verbal association states revealed that the two measures are isomorphic with respect to individual differences. Furthermore, the relationship between human-rated originality scores in typical association condition and the entropy difference between original and typical association states is small and negative, but statistically not significant. A significant negative correlation would suggest that individuals with a larger MSE difference make less original associations when this is their task. However, the relationship of the MSE difference with originality scores in the original association condition was positive. This means that individuals who show a larger difference in small-scale entropy between the original and typical associations are better able to conform to the requirements of both tasks. These associations, however, need further investigation because none of these correlations could be robustly established with the data at hand. Taken together, we report results that are partly in line with the hypothesis that BSC is a sensitive neural marker of creative verbal association generation.

Higher entropy during original association production as compared with typical associations shows that signal complexity is a sensitive average marker of verbal creativity. This means that brain activity tends to become more complex in average when producing original verbal associations. This main finding can be considered a step ahead to establish brain complexity as a correlate of creative verbal associations. It goes beyond previous studies that solely associated resting BSC as a trait measure with creativity task performance. For example, Ueno et al. (2015) found that more creative elderly individuals exhibited higher MSE measured in resting-state EEG. To our best knowledge, only the study by Rominger et al. (2019) examined creative idea production as reflected in EEG signals during a creative task (figural DT) and showed increased functional coupling of brain networks from idea generation to idea elaboration. Complementing these previous studies, the present study is a further step toward an elaborated neural complexity theory of creativity.

Since both brain oscillations and MSE characterize the dynamical features of a time series, one may wonder what the conceptual differences between these two measures are. And how do they differentially reflect the functional characteristics of the brain? Some studies have related creativity with neural oscillations (for a review, see Fink and Benedek, 2014). Neural oscillations also provide critical information about neural dynamics. For example, slow neural oscillations reflect mechanisms that support information integration and communication between large-scale neural networks (Cohen, 2014). Thus, oscillation measures (e.g., power spectra of different frequency bands) are also suitable for studying brain activities over multiple spatiotemporal scales. However, conceptually, oscillation measures are different from MSE measures. By definition, oscillation reflects the predictable feature of the dynamics and may be too simple when high level cognition like creativity is concerned. Following the Honing theory, creativity is assumed to be associated with higher psychological entropic states, characterized by large-scale functional and connectivity patterns reflecting complex dynamical interacting systems (Zabelina and Andrews-Hanna, 2016; Beaty et al., 2018). Therefore, we need a suitable method to identify this entropic state and parameterize the complexity over multiple timescales. As elaborated when introducing the MSE algorithm, small and medium MSE timescales reflect fast and local neural dynamic activities, and large scales are concerned with slow dynamics across broader spatial scales. On this account, we propose that MSE is a conceptually more suitable measure to parameterize the complexity of creative brain activity.

As predicted, the higher complexity during original associations was highest in frontal regions. Our results thus add up to the knowledge on the involvement of frontal areas during creative tasks. For example, Dietrich and Kanso (2010) summarized the literature according to which vast areas of the PFC are consistently involved during performance on different creativity tasks. Further empirical evidence has also revealed an active anterior PFC, especially during creative idea generation, musical improvisation, analogical reasoning, and metaphor processing (Abraham, 2018).

With this study, we aimed to go beyond mean differences findings by additionally demonstrating specific rank orders of individuals in less vs. more creative brain states. However, we found no specificity. This means that entropy is quantitatively higher during original verbal production as compared with typical verbal production, but it does not differentiate individuals depending on their brain states. Thus, there is a shift in mean MSE depending on the brain state, but individual differences in MSE remain stable across states. A possible explanation for the non-expected perfect rank order stability finding can be that both cues (original and typical associations) tap into the lexical system, where as a consequence of activating a concept (by the noun) an associated word (the verb) is produced. If an individual has a rich lexicon, she/he will be able to produce highly original verbs (semantically distant or indirectly associated with the noun) relying on the same entropic brain state needed to activate the most conventional associations. In these individuals, the difference in entropy between the two conditions might be larger than in individuals with a poor lexicon, who will be less able to find original as well as typical associations.

Another potential explanation of these findings is that MSE might not be sensitive enough to differentiate the closely coupled dimensions of creative ability—theoretically typical verbal production being considered as the fluency facet of creativity. Therefore, during an attempt to produce original associations over several seconds, these closely related facets of creative ability should probably be rapidly shifting back and forth. Because MSE analysis integrates over larger time intervals, i.e., several seconds that leads to low time resolution, such brief creative states (producing typical and original associations) in the brain become inseparable when such larger swaths of time are considered.

## Future Directions

The current findings are limited to a word production task that taps into one aspect of creativity, i.e., verbal creativity. Future studies focusing on individual differences in creativity will need to employ task(s) that can capture multiple aspects of creativity (i.e., fluency, flexibility, and originality, also in figural and numerical domains). A new line of creativity studies recently proposed a neurocognitive framework of creative cognition that should be characterized as an interplay between memory, attention, and cognitive control (Benedek and Fink, 2019). In addition, resting-state functional connectivity in cognitive control networks has been shown to be associated with creativity (Beaty et al., 2014, 2018; Sun et al., 2019). Therefore, in future complexity studies on creative cognition, it will be critical to co-examine specific cognitive functions elementary to creative cognition. Thus, a larger psychometric task battery including cognitive control, working memory, verbal knowledge tasks, and resting-state brain activity would increase sophisticated understanding of creative brain states in terms of individual differences. Furthermore, recently proposed methods for explicit identification of multivariate patterns in neural data (Haxby et al., 2001; Fahrenfort et al., 2018) could be combined with entropy estimates in the future. The aim would be to measure the transition among the identified multivariate patterns as a potential marker to quantify the spatiotemporal switching of the dynamical patterns, which may allow better differentiating creative vs. less-creative states. Because MSE captures complexity across different temporal scales only, it can just implicitly reflect the spatiotemporal interactions in the underlying neural systems (Liu et al., 2019). In summary, future studies might successfully combine modern brain signal analysis methods with multivariate modeling of brain-behavior associations to better understand individual differences in verbal creativity.

## Data Availability Statement

All datasets generated for this study are included in the article/Supplementary Material.

## Ethics Statement

The studies involving human participants were reviewed and approved by the ethics committee of the Department of Psychology, Humboldt-Universität zu Berlin (approval number 2012-46). The patients/participants provided their written informed consent to participate in this study.

## Author Contributions

YK and AH conceptualized the study. YK designed it, collected the data, which she independently preprocessed and analyzed, discussed results with all co-authors, and drafted the manuscript. AH and WS supervised the task design and the EEG data acquisition, processing and statistical analysis, and results interpretation and theoretical discussion. GO and CZ supervised the MSE analysis and contributed to the interpretation of results. SW contributed to the behavioral data collection and analysis. All co-authors were involved in the editing of the manuscript at several stages.

## Conflict of Interest

The authors declare that the research was conducted in the absence of any commercial or financial relationships that could be construed as a potential conflict of interest.
