# Supplementary material for: What Does Temporal Brain Signal Complexity Reveal About Verbal Creativity?
Source: Front Behav Neurosci. 2020 Aug 27;14:146. doi: 10.3389/fnbeh.2020.00146 (PMC7481454; doi:10.3389/fnbeh.2020.00146)
Supplement: Supplementary file 3 [file Data_Sheet_1.docx]

**Supplementary Materials**

**S1**

We adapted the original Verb generation task (Prabhakaran, Green, and Gray, 2014). The following items were presented in the main trials which were translated from English to German language. Highlighted items were dropped in the MSE analysis part due to language translation problem. For example, ‘sofa’ and ‘couch’ in German language are synonyms; same reason applies for ‘säugling’ and ‘baby’. And the item ‘zu hause’ was not properly translated for the noun ‘home’, and hence was dropped from the analysis.

| Items | English Verison | German Version |
| --- | --- | --- |
| 1 | Golf | Golf |
| 2 | Church | Kirche |
| 3 | House | Haus |
| 4 | Tree | Baum |
| 5 | Clay | Lehm |
| 6 | Infant | Säugling |
| 7 | Lamp | Lampe |
| 8 | Office | Büro |
| 9 | Cannon | Kanone |
| 10 | Key | Schlüssel |
| 11 | Taxi | Taxi |
| 12 | Razor | Rasierer |
| 13 | Blade | Klinge |
| 14 | Hair | Haare |
| 15 | Fist | Faust |
| 16 | Shoe | Schuh |
| 17 | Drug | Droge |
| 18 | Poem | Gedicht |
| 19 | Finger | Finger |
| 20 | Birth | Geburt |
| 21 | Horn | Horn |
| 22 | Boot | Stiefel |
| 23 | Artist | Künstler |
| 24 | Soup | Suppe |
| 25 | Rock | Stein |
| 26 | Bucket | Korb |
| 27 | Pan | Pfanne |
| 28 | Couch | Couch |
| 29 | Soap | Seife |
| 30 | Candle | Kerze |
| 31 | Ship | Schiff |
| 32 | Cart | Karren |
| 33 | Coal | Kohle |
| 34 | Dish | Geschirr |
| 35 | Rose | Rose |
| 36 | Tool | Werkzeug |
| 37 | Flower | Blume |
| 38 | Drum | Schlagzeug |
| 39 | Muscle | Muskel |
| 40 | Canoe | Kanu |
| 41 | Card | Karte |
| 42 | Pill | Tablette |
| 43 | Tune | Melodie |
| 44 | Needle | Nadel |
| 45 | Feet | Füße |
| 46 | Belt | Gürtel |
| 47 | Glass | Glas |
| 48 | Store | Laden |
| 49 | Coffee | Kaffee |
| 50 | Pillow | Kissen |
| 51 | Money | Geld |
| 52 | Leaf | Blätter |
| 53 | Ring | Ring |
| 54 | Sofa | Sofa |
| 55 | Manual | Handbuch |
| 56 | Bread | Brot |
| 57 | Baby | Baby |
| 58 | Street | Straße |
| 59 | Snow | Schnee |
| 60 | Shovel | Schaufel |
| 61 | Phone | Telefon |
| 62 | Oven | Ofen |
| 63 | Grass | Gras |
| 64 | Paper | Papier |
| 65 | Home | zu Hause |
| 66 | Debt | Schulden |
| 67 | Note | Notiz |
| 68 | Letter | Brief |
| 69 | Hole | Loch |
| 70 | Oath | Eid |
| 71 | Tongue | Zunge |
| 72 | Music | Musik |
|  |  |  |
|  |  |  |

Figure S1: Distribution of average response times (Avg RT) in seconds across all participants which was required to produce typical and original associations. Different colors of the bars represent frequency of the data.


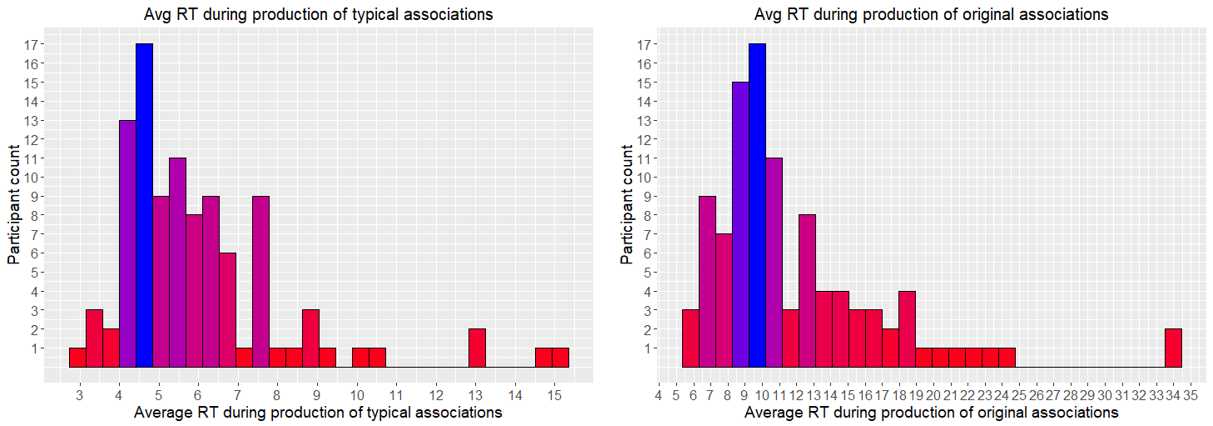


**S2**

Correlation matrices between four different segments of the MSE values for standard length of the trials and varied trial length for frontal electrodes and 5,10,15, and 20 time scales. Dark brown areas show stronger correlation between the different segments which shows that the standardized length or variable length of the trials do not make difference. Hence, in order to keep the maximum trial length, we select varied trial length for all of the datasets.


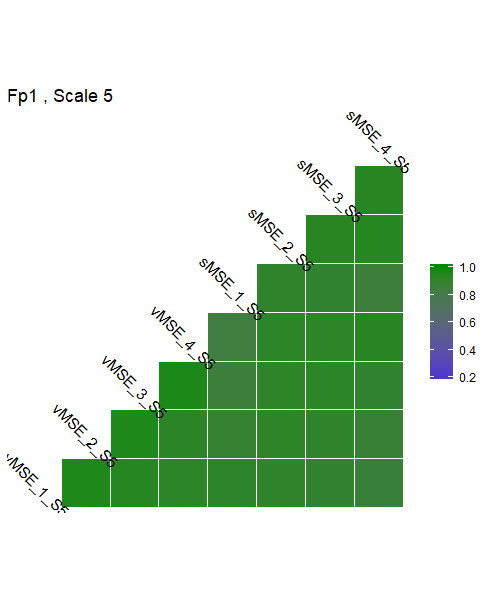

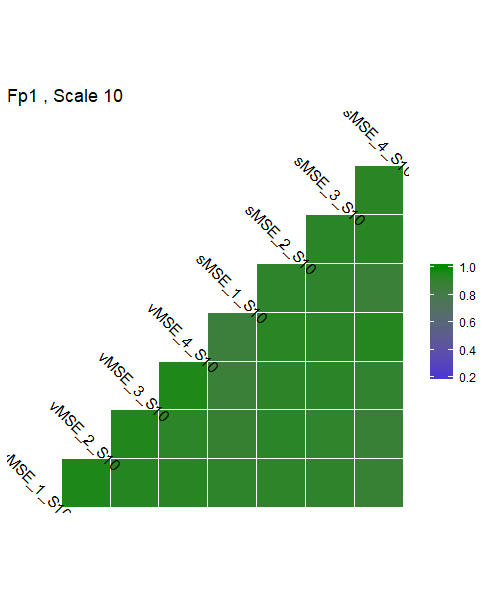

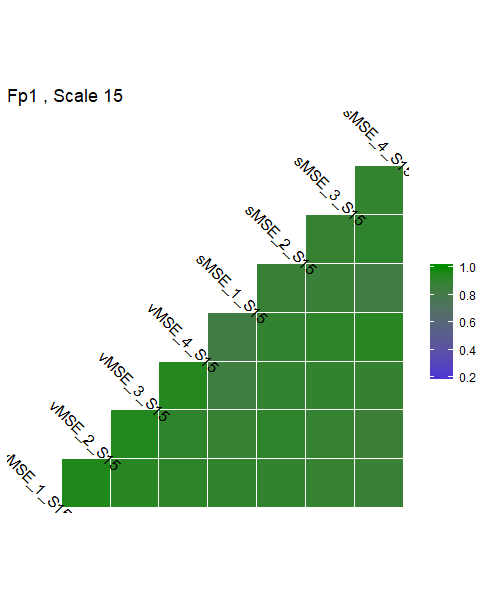

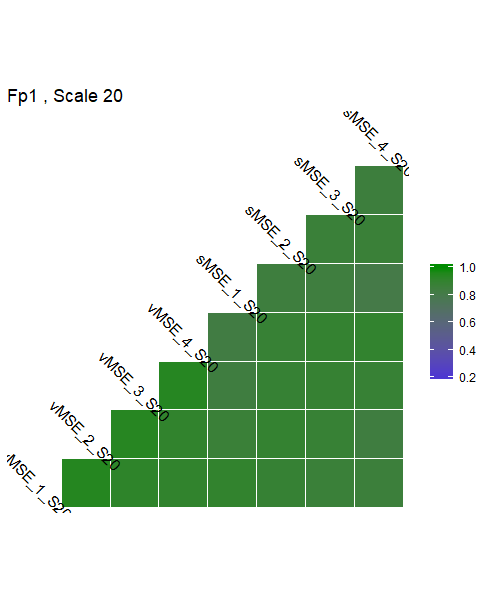

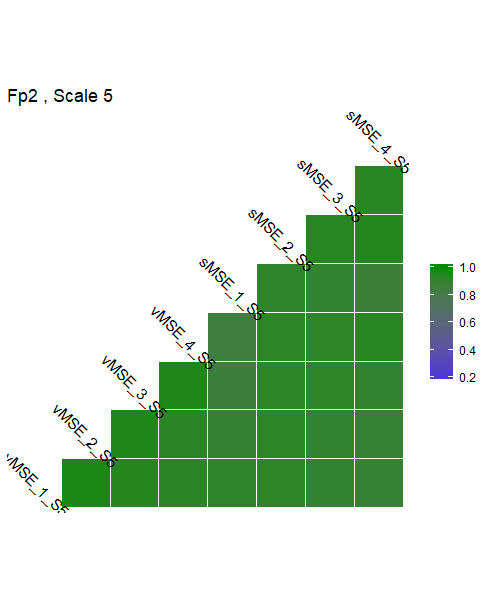

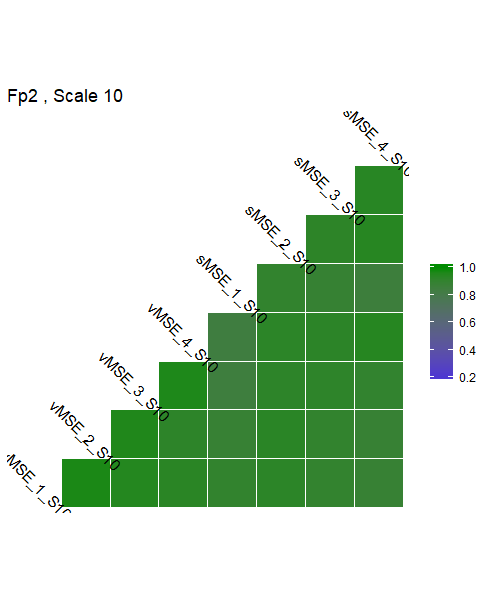

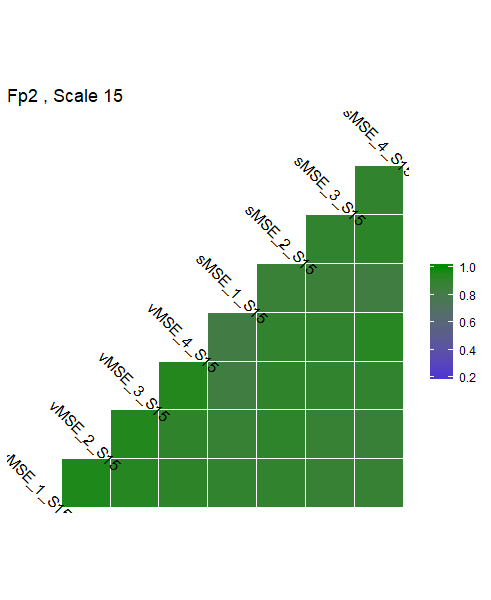

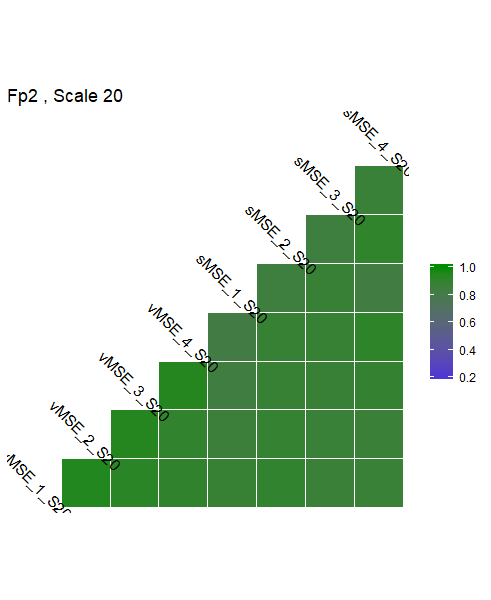

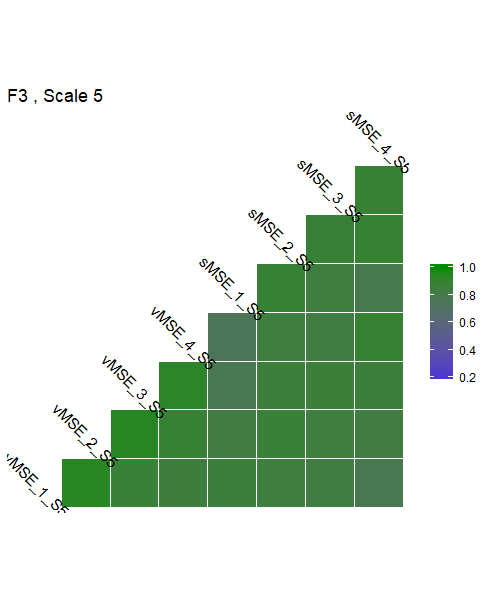

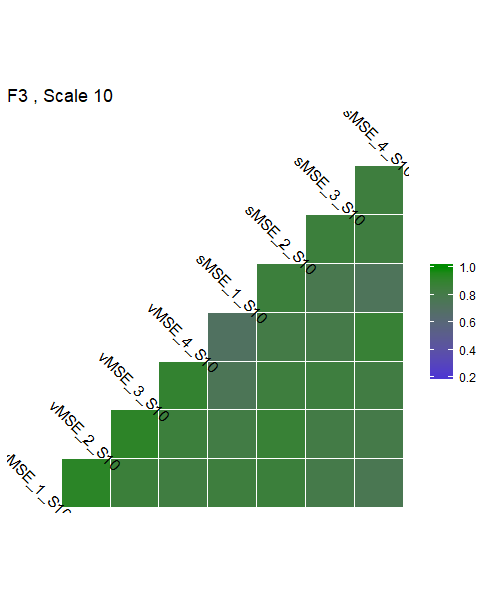

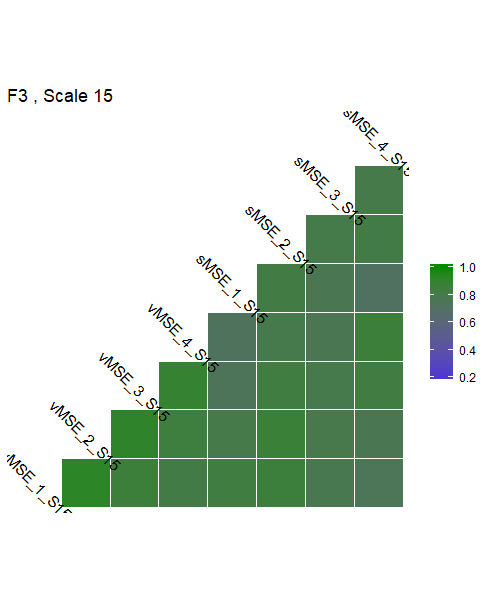

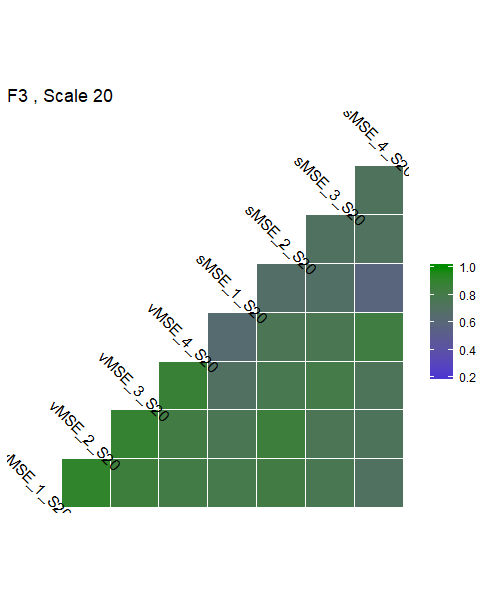

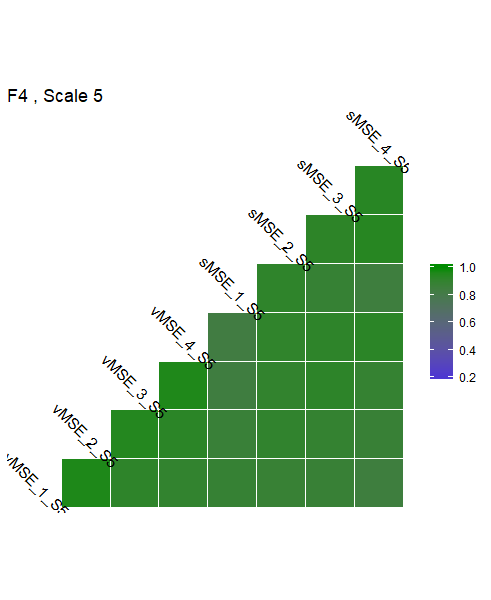

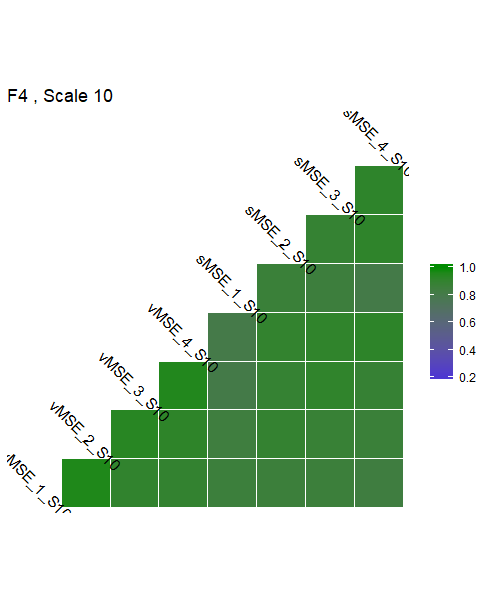

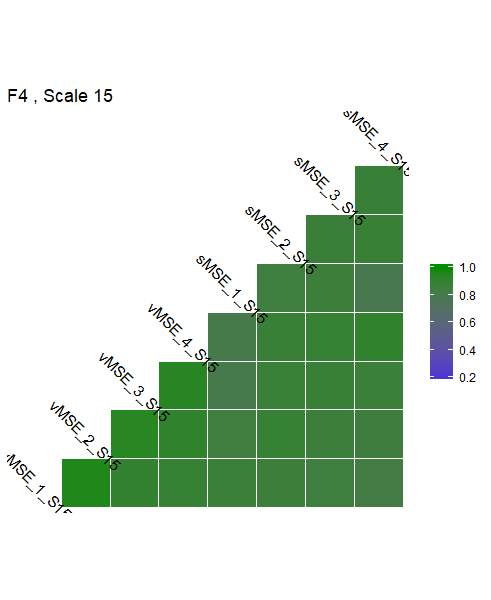

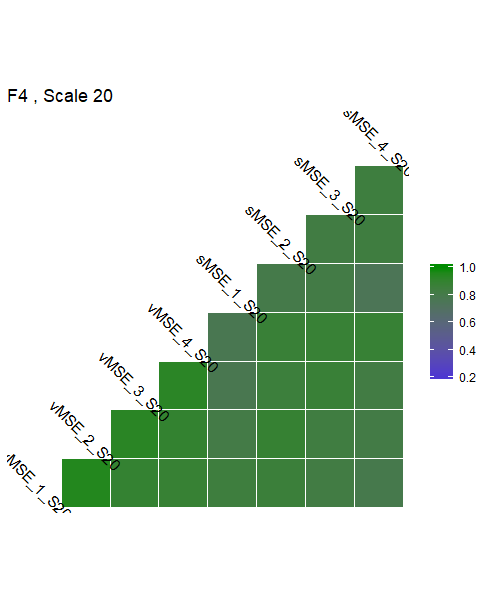

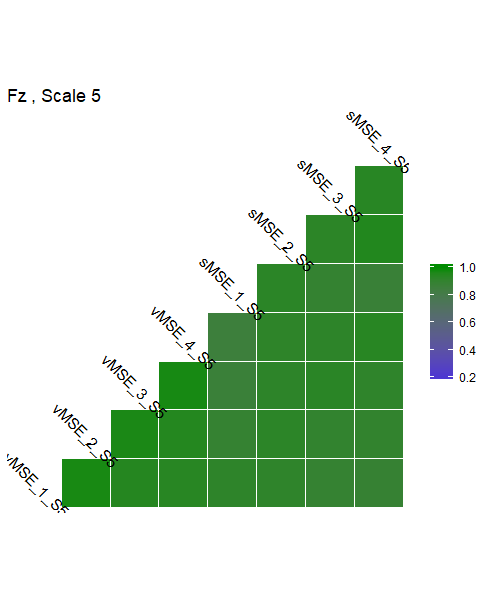

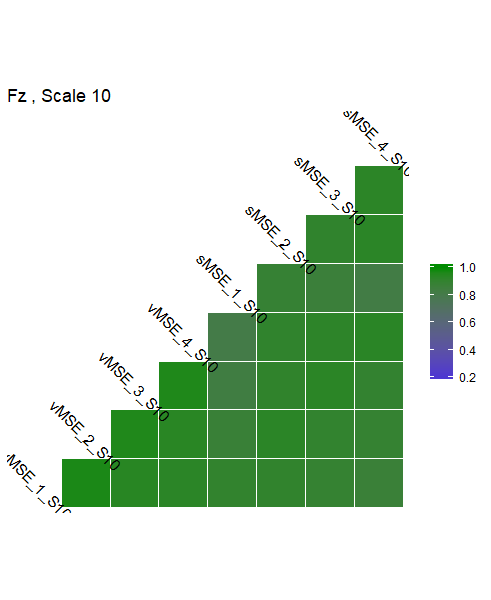

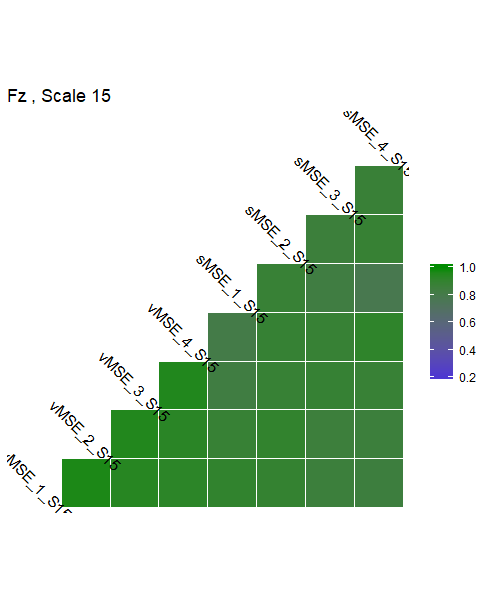

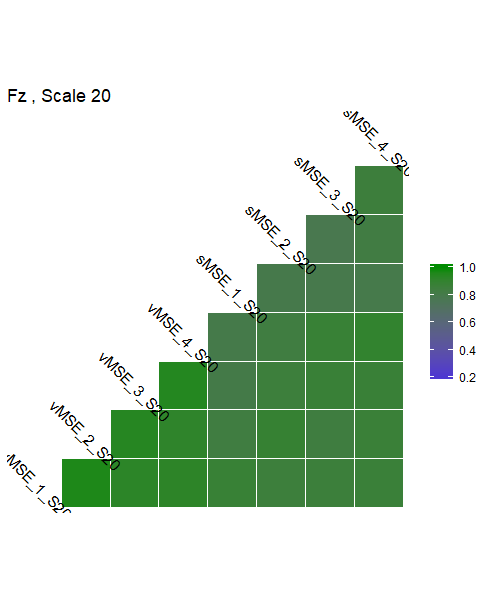


**
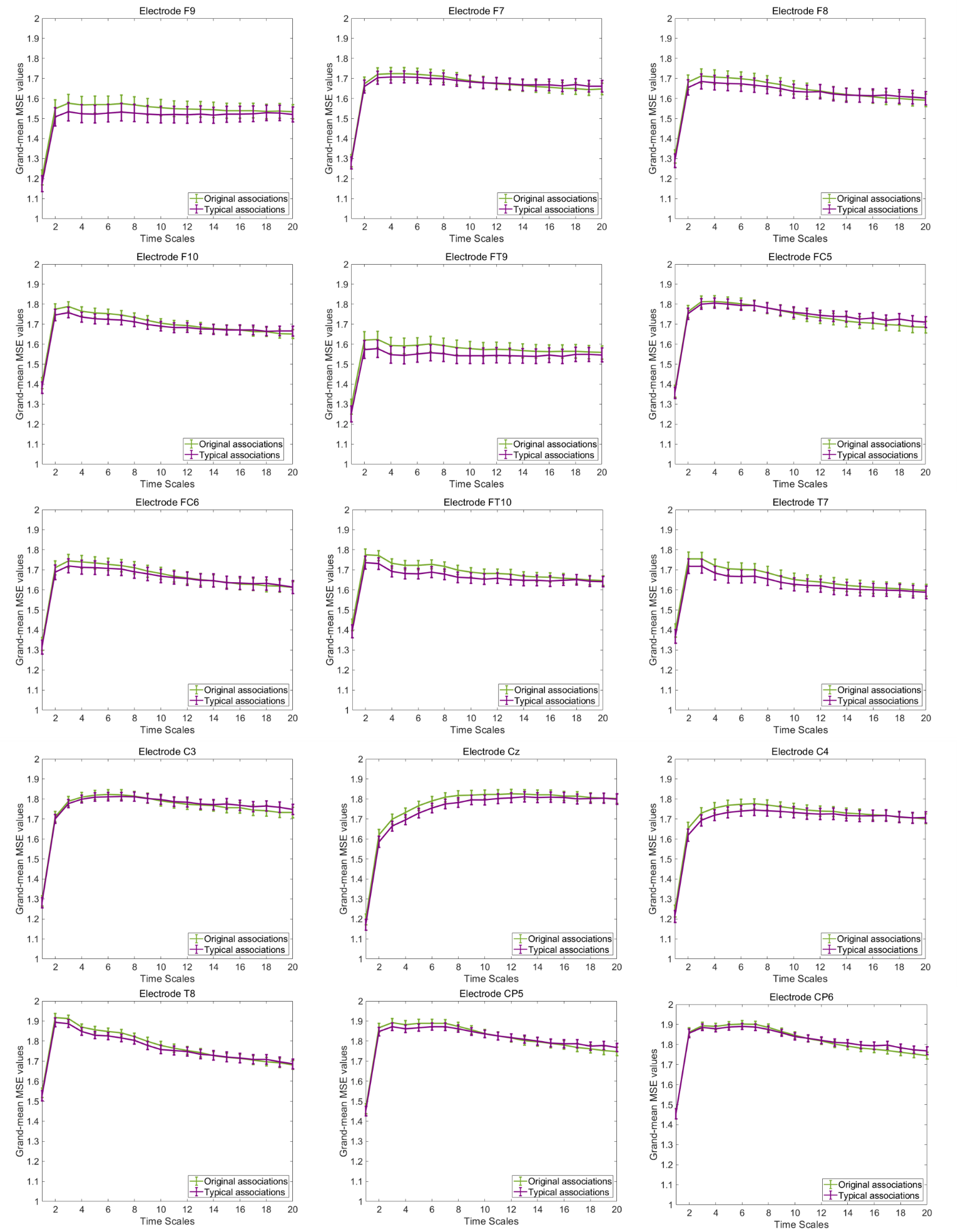
S3**


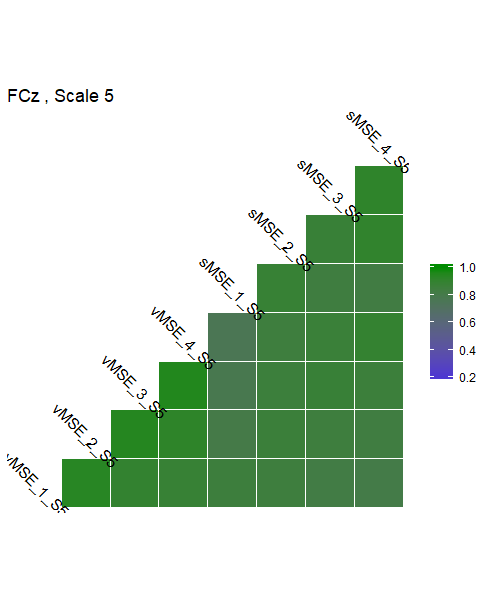

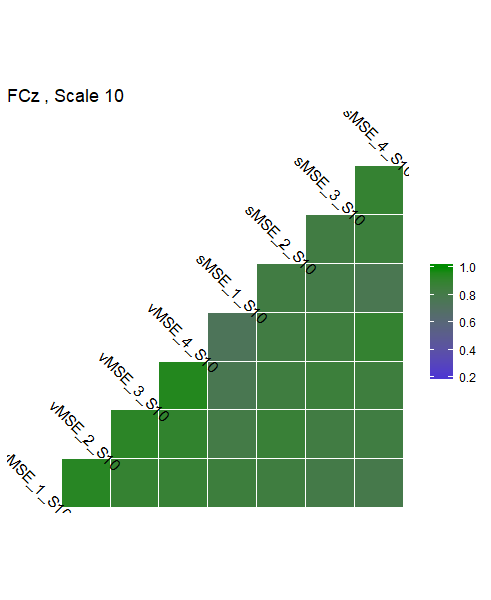

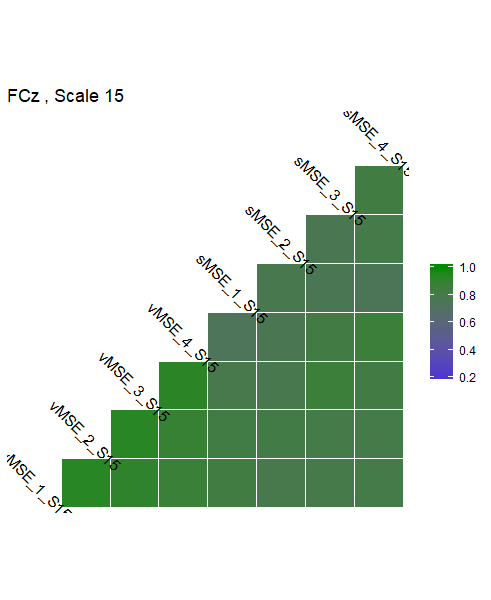

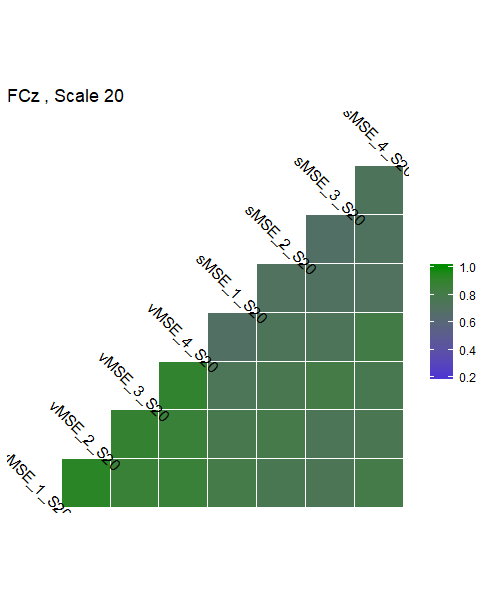


Figure S2: Correlation matrix plots between standard length and variable length MSE segments. vMSE_1_S5 represents first segment of MSE for scale 5; sMSE_2_S10 represents second segments of MSE for scale 10. Correlation between different lengths of MSE are stronger (<=.70).

Figure S3: Grand-mean MSE during typical and original associations at remaining 24 electrodes across 20 time scales. The MSE is slightly larger in the originality condition mostly at frontal electrodes at small (1-5), and medium (6-15) time scales but show opposite pattern in parietal electrodes at higher scales. Error bars represent 1 SE.


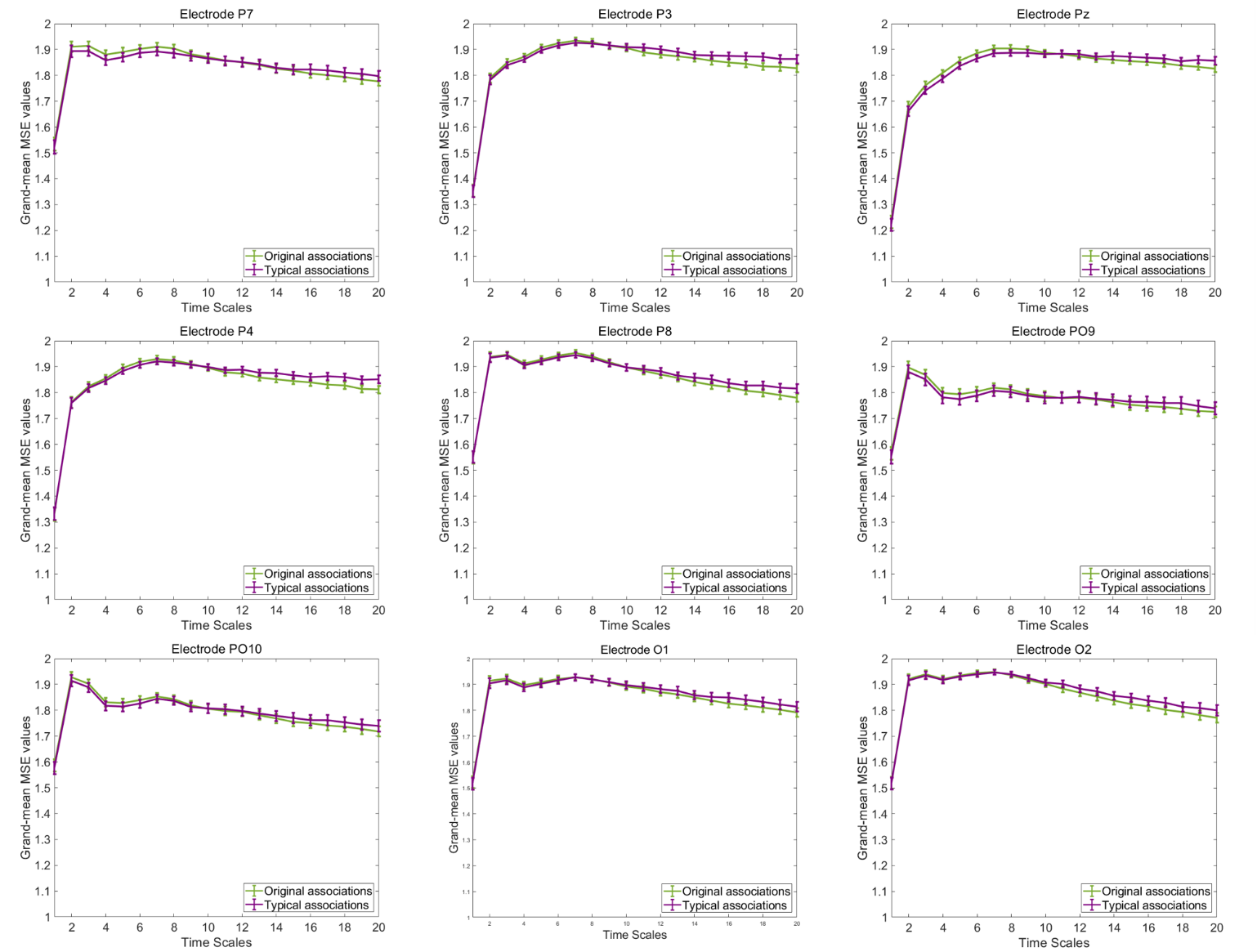


**S4**

Figure S4: Mean differences in MSE in original and typical associations computed using *t*-test showing no statistically substantial higher mean MSE in females as compared to males in none of the experimental conditions. Red color shows MSE in females and blue signifies males.The reults show that females tend to have higher mean MSE in both original and typical associations but none of the results are statistically significant.Therefore, there are no sex differecnes in MSE in the two experimental conditions.


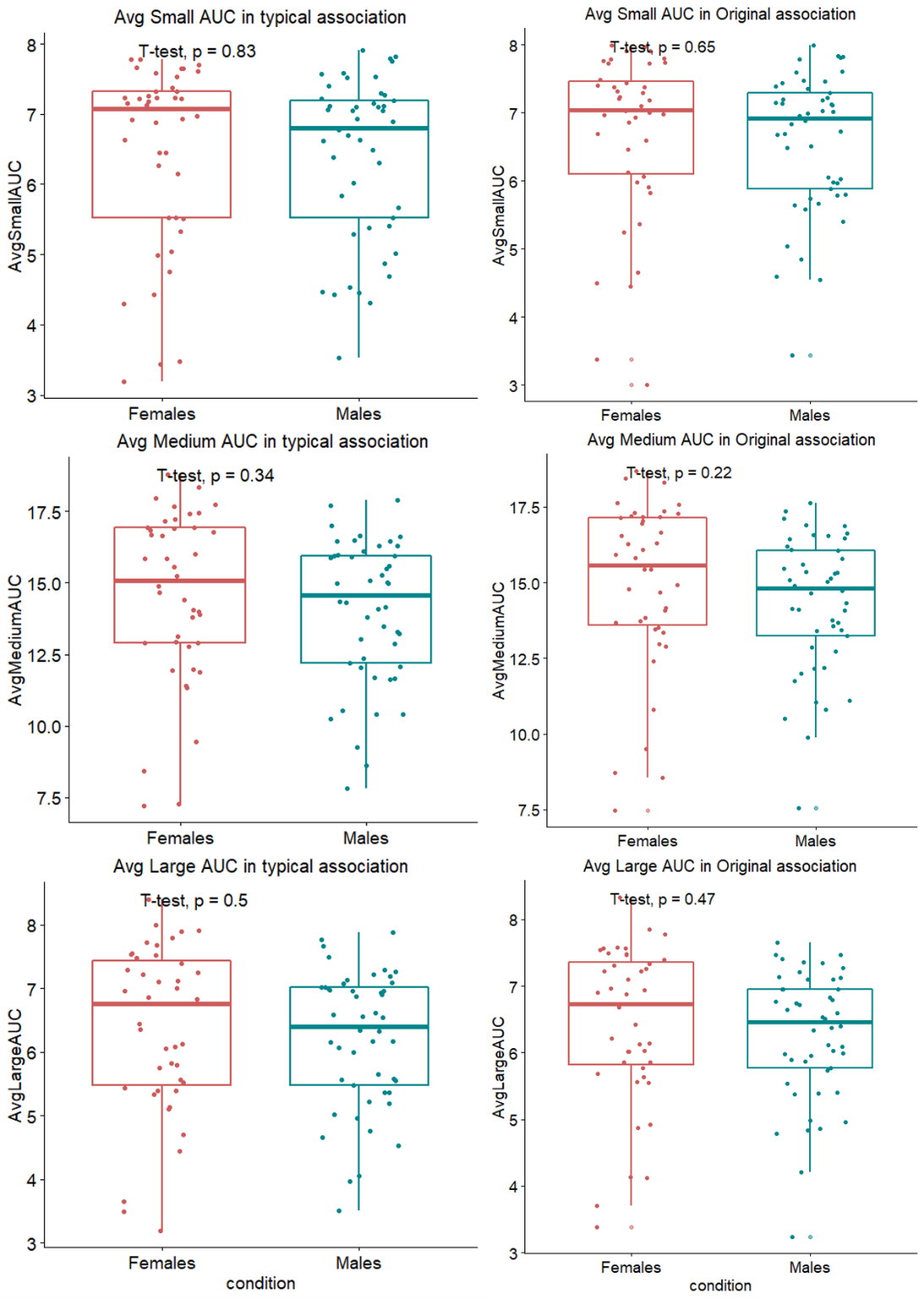


**S5**


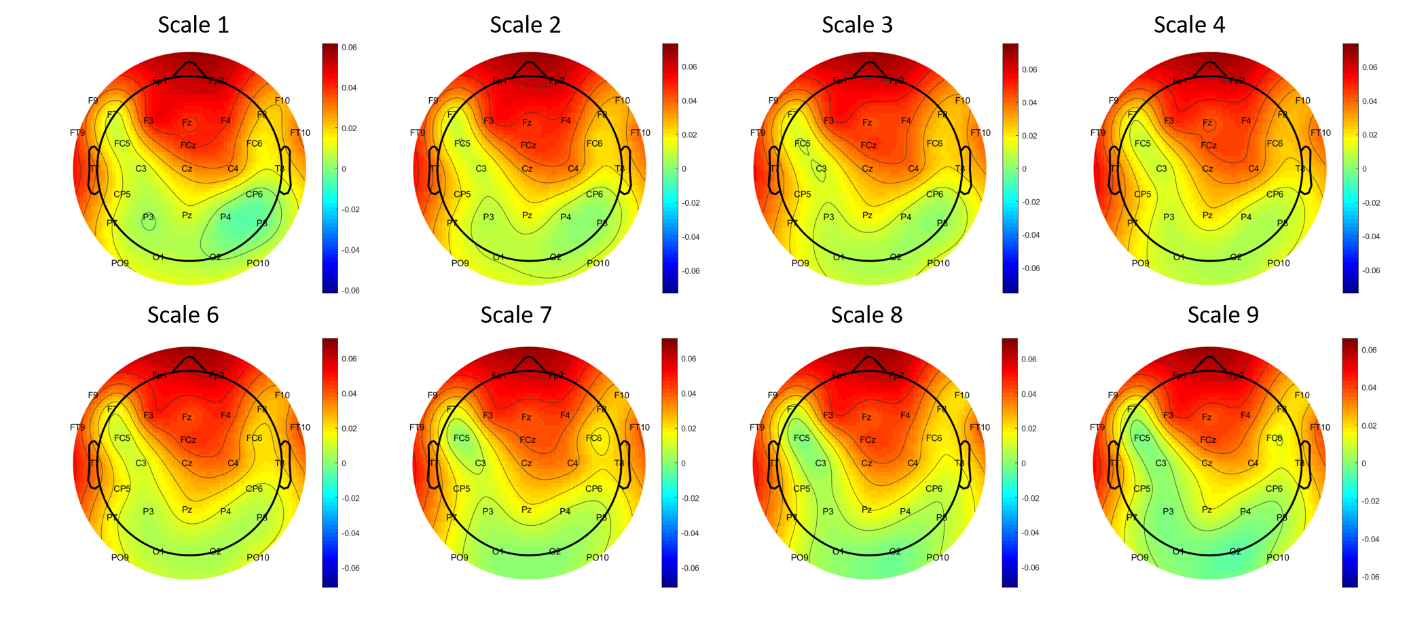

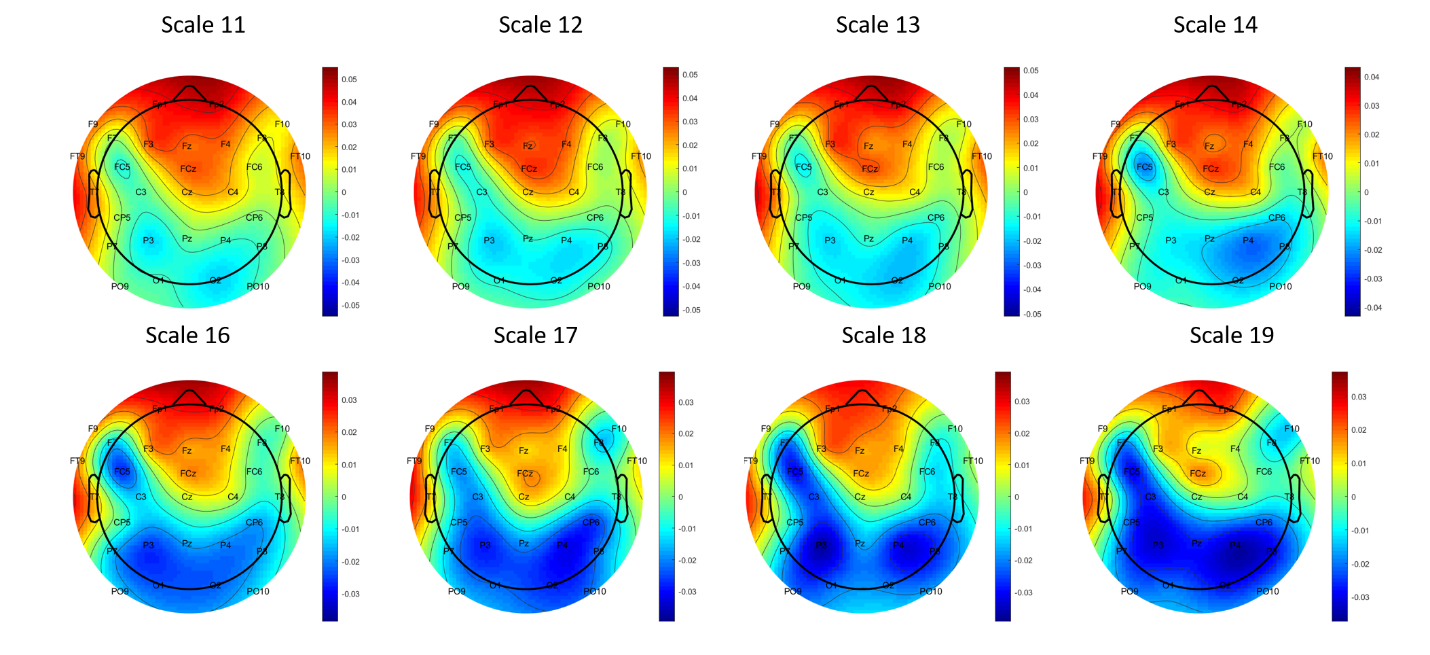


Figure S5: Topographic plots of grand-mean MSE based on average MSE difference values between original and typical association across the temporal scales from 1-4,6-9,11-14, and 16-19. Together with Fig. 5 for temporal scale 5, 10, 15 and 20, these distributions illustrate that the positive difference in MSE between the two-word generation conditions occur at frontal regions and the negative difference is prominent in parietal regions.

| Model | Correlation of the difference score with human rated originality scores during | |
| --- | --- | --- |
|  | Typical associations | Original associations |
|  |  |  |
|  |  |  |
| Small Parietal AUC | -0.054 (*p*=0.7) | 0.206(*p*=0.1) |
|  |  |  |
| Medium Parietal AUC | 0.013 (*p*=0.9) | 0.115 (*p*=0.5) |
|  |  |  |
| Large Parietal AUC | 0.185 (*p*=0.4) | -0.206 (*p*=0.3) |
|  |  |  |

**S6**

Table S6: Relationship between the difference scores for AUC values calculated for parietal ROIs and human rated originality scores in typical and original associations.
